# 

**Published:** 1867-08

**Authors:** 


### THE CHICAGO MEDICAL JOURNAL.

**Edited by J. ADAMS ALLEN, M.D., LL.D.**

Prof. Principles and Practice of Medicine and Clinical Medicine in Rush Med. College.
All letters for the JOURNAL should be addressed to DR. J. ADAMS ALLEN, P.O. Box 1948, Chicago, Ill.

**TERMS---$2 PER ANNUM, IN ADVANCE.**

$3 if not paid before 1st of July in each year. Single copies, 25 cents.

